# Lifestyle habits and obesity indices among male adolescents in Riyadh, Saudi Arabia

**DOI:** 10.1038/s41598-023-37920-5

**Published:** 2023-07-26

**Authors:** Abdulrahman Mohammed Alhowikan, Nagwa Ebrahim Alsharqawy, Nouf Nawaf Alazmaa, Abdullah Ibrahim Saeed, Yasser Abdullah Alhazzani, Nasser Yousef Alhowaish, Abdulaziz Fahad Bin Yahya, Zeyad Ahmed Alhozaimi, Naif Eid Aleid, Maha H. Alhussain

**Affiliations:** 1 Department of Physiology, College of Medicine, King Saud University, Riyadh, Saudi Arabia; 2 Faculty of Arts Social Studies, King Saud University, Riyadh, Saudi Arabia; 3 National Unified Procurement Company NUPCO, Riyadh, Saudi Arabia; 4 Department of Family Medicine, College of Medicine, King Saud University, Riyadh, Saudi Arabia; 5 Department of Food Science and Nutrition, College of Food and Agriculture Sciences, King Saud University, Riyadh, 11451 Saudi Arabia

**Keywords:** Nutrition, Public health, Quality of life

## Abstract

Obesity among adolescents is a global health apprehension which requires early prevention. The aim of this study was to determine the association between lifestyle habits including physical activity, sedentary behaviors and eating habits with obesity indices of body mass index (BMI) and waist-to-height ratio (WHtR) among male adolescents in Riyadh, Saudi Arabia. We randomly selected 471 secondary school male adolescents aged 14–18 years. A pre-validated self-reported questionnaire was used to record the data on physical activity level, sedentary behaviors, sleep duration and eating habits. The International Obesity Task Force (IOTF) cutoff values for adolescents under 18 years of age were used to define overweight and obesity. Total energy expenditure was calculated using metabolic equivalent-minutes per week. Anthropometry including weight, height, BMI, waist circumference, waist/height ratio (WHtR), were assessed. 53.7% and 48.4% of the adolescents were overweight/obese and had abdominal obesity; respectively. Those with overweight and obesity or above 50% of WHtR were much less active in terms of METs-min/week from vigorous-intensity sports, sum of all METs-min/week from all vigorous-intensity physical activity, total METs-min/week from all physical activity compared with non-obese adolescents and below 50% of WHtR. The present study identified the lifestyle habits that were associated with obesity and may represent valid targets for the prevention and management of obesity among Saudi adolescents. Knowledge of the factors that contribute to obesity could be used in preventive programs for the control of obesity among adolescents.

## Introduction

Obesity is a chronic condition characterized by an excessive accumulation of body fat and increases the risk of numerous medical conditions including type 2 diabetes mellitus hypertension, coronary artery disease and certain type of cancer^1^. Obesity has increased dramatically^2^, and the global prevalence of obesity has become a health problem affecting young people from low-income, middle-income and high-income countries^3^. According to the World Health Organization (WHO), over 340 million children and adolescents aged 5–19 were overweight or obese in 2016^4^.

In recent years, adolescents’ obesity is becoming a serious public health problem in many Arab countries including Saudi Arabia^5,6^. The increasing prevalence of adolescent obesity is associated with a rise in comorbidities^7^. It has been reported that an overweight child is 77% more likely to be an obese adult and develop a risk of high blood pressure, hyperlipidemias, coronary artery diseases, diabetes mellitus with negative impact on physical, social and cognitive functions^8–10^. The Global Burden of Disease study reported obesity was the key risk factor for disability-adjusted life years in of Saudi Arabia^11^. These emphasize the need for effective strategies to tracle the increased prevalence of obesity and associated complications.

Obesity is a multi-factorial issue that interweaves genetic, biological, psychological, environmental and lifestyle factors^7,12^. It is well stablished that unhealthy lifestyle habits including sedentary behavior, physical inactivity and poor dietary habits are linked to obesity. In Saudi Arabia, although studies have shown increased prevalence of overweight and obesity among adolescents^13–15^, literature assessing the relationships between obesity and habitual lifestyle is lacking. Therefore, the present study aimed to evaluate the association between lifestyle habits including physical activity, sedentary behaviors, eating habits and sleep duration with obesity indices among Saudi male adolescents.

## Methodology

### Study population

This study is the part of Arab Teens Lifestyle Study (ATLS), the school-based cross-sectional multicenter collaborative study^16^. The study subjects were Saudi male adolescent recruited from the secondary schools in the capital city of Riyadh, Saudi Arabia. Subjects were selected using a cluster sampling practice. Inclusion criteria were as follows: healthy students aged 14–18 years enrolled in secondary schools. Exclusion criteria were students with any physical impairment. The sample size was calculated based on the population of male students in public and private secondary schools in Riyadh (about 75,000). Within ± 0.05 of the population proportion and a 95% confidence level, the required total sample size was 382 students. Additional students were included to compensate for missing data. A total of 471 students aged 14–18 years were included in the study.

The study was conducted according to the guidelines established by the Declaration of Helsinki. The study was approved by the Ethical Review Committee, College of Medicine, King Saud University, Riyadh (Ref. No. 15/0531/IRB) and the General Directorate of School Education in Riyadh. All the schools and students consented to participate in the study. Informed consent was obtained from all the parents/legal guardians of the subjects.

### Anthropometric measurements

Height was measured to the nearest 0.1 cm using a calibrated measuring rod, while the subject was in a full standing position and without shoes. Body weight was measured to the nearest 0.1 kg while subjects were wearing light clothing with no shoes and with an empty bladder using a calibrated portable medical scale (Seca, Germany) placed on a hard-flat surface and with the digital screen indicating zero and subjects were instructed to stand on the scale with both feet Body mass index (BMI) was calculated according to the standard formula, the ratio of weight in kilograms by the height squared in meters (kg/m^2^). Waist circumference was measured using a tape measure at the midpoint between the lower margin of the last palpable rib and the top of the iliac crest and the zero end of the tape was place below the section containing the measurement value. The tape was put parallel to the floor and was not compress the skin. Hip circumference was measured in a horizontal plane at the point yielding the maximum circumference over the buttocks. The waist-to-hip ratio was calculated as the ratio of the waist and hip circumferences. All measurements were measured according to the standard protocols^17^ and were performed by a trained investigator.

The International Obesity Task Force (IOTF) age and sex specific BMI cutoff reference standards were used to classify adolescents with obesity or overweight between the ages of 14–17 years^18^. For those who aged 18 years and older, we used WHO adult cutoff points of 25–29.9 kg/m^2^ to define overweight and 30 kg/m^2^ and higher for obesity. Waist height ration (WHtR) were calculated and WHtR cut-off point of 0.50 was used to define abdominal obesity^19^.

### Study questionnaire

The Arab Teen Lifestyle Study (ATLS) questionnaire^16^ was used to record a number of lifestyle variables and it comprised 47 items, related to patterns of physical activity, sedentary activity, sleep duration and eating habits. ATLAS questionnaire has previously been demonstrated to be reliable and valid to assess physical activity and lifestyle habits among youth aged 14 to 25^16,19,20^.

### Physical activity

Questions on physical activity were part of the ATLS questionnaire. Physical activity allied questionnaire included the information on the frequency, duration and intensity of light, moderate and vigorous intensity physical activities during the week. The questionnaire covers various areas including fitness, sport and leisure-time activities. In addition, the questionnaire included items about when, where and with whom the participant exercise, active or inactive. For the determination of subjects’ levels of physical activity, we used the total energy expenditure in METs-min per week spent in all physical activity, sum of energy expenditure in METs-min per week spent in all moderate intensity physical activity and sum of energy expenditure in METs-min per week spent in all vigorous intensity physical activity. Moreover, physical activity was categorized into active or inactive level based on below or above 1680 METs-min/week.

### Sedentary behaviors and sleep duration

The ATLS questionnaire includes questions related to sedentary behaviors and sleep duration. Students reported on the typical time in hours spent per day on screen activities, including television viewing (TV), video games, computer and recreational internet use during week days and weekends. We used a cut off value of below or above 3 h for total daily time spent in screen. Sleep durations in hours spent on weekdays and weekends, the cut-off hours used for sufficient or insufficient sleep duration was set at below or above 8 h per day^21^.

### Eating habits

Eating habits were recorded based on the eating habits during the week was a segment of ATLS questionnaire. Participants reported times per week they consume breakfast, vegetables (cooked and uncooked), fruits, milk and dairy products, sugar-sweetened drinks (including soft drinks), fast foods, donuts/cakes, sweets and chocolates and energy drinks.. The students were given a choice of answers, ranging from zero intake (never) to a maximum intake of 7 days per week (every day). Dietary intake frequencies were categorized into three categories: 1–2 days, 3–4 days and 5 or more days per week.

### Data and statistical analysis

Data were entered into a coded SPSS entry sheet, checked, cleaned and analyzed using IBM SPSS software, version 22. To guard against over reporting, the total time spent at all types of physical activity was capped at 28 h per week (4 h per day). Time spent in all screen was also capped at a maximum of 16 h per day. Descriptive statistics were calculated and presented as means and standard errors. In addition a multivariate analysis was used to test the differences between overweight/obesity and WHtR categories in selected lifestyle habits. Chi Square test for observed versus expected frequency was also used to test differences in some of the variables. Finally, we used multiple linear regression analyses to predict total activity energy expenditure in METs-min/week and sum of all METs-min/week from all vigorous-intensity physical activity with stepwise method. Entered predictors included age, BMI, waist circumference, WHtR, average screen time, average sleep duration, breakfast intake, vegetables intake, fruit intake, milk/dairy products intake, sugar-sweetened drinks intake, fast food intake, French fries/potato chips intake, cake/donuts intake, chocolate/candy intake. The level of significance was established at *p* < 0.05.

## Results

Descriptive characteristics of the study sample are shown in Table 1. Overall, approximately half (53.7%) of the sample were either overweight or obese. As expected, overweight/obese students had significantly greater weight, BMI, waist circumference and WHtR than non-overweigh/non-obese students.

**Table 1:** Descriptive characteristics of the students.

Variable	All (N = 471)	Non-overweigh/non-obese (N = 218)	Overweight/obese (N = 253)	*p* value *
Age (years)	17.0 (0.04)	17.1 (0.06)	16.9 (0.06)	**0.040**
Weight (kg)	75.5 (0.95)	59.1 (0.94)	89.5 (0.87)	**< 0.001**
Height (cm)	170.4 (0.31)	169.8 (0.45)	170.9 (0.42)	0.071
BMI (kg/m^**2**^)	25.9 (0.31)	20.5 (0.29)	30.6 (0.27)	**< 0.001**
Waist circumference (cm)	86.2 (0.74)	73.6 (0.75)	97.1 (0.69)	**< 0.001**
WHtR	0.51 (0.004)	0.44 (0.005)	0.57 (0.004)	**< 0.001**
Overweight and obesity, based on BMI cut-offs (%)	53.7	–	–	–
Abdominal obesity, based on WHtR ≥ 0.50 (%)	48.4	–	–	–

Significant values are in [bold].

*WHtR* Waist/height ratio.

Data are means ± standard errors.

*Multivariate analyses test results.

Table 2 presents the results of activity energy expenditure (METs-minutes/week) expended in several types of physical activity relative to overweight/obesity and waist/height ratio categories. Overweigh/obese students were more sedentary and much less active in terms of METs-min/week from vigorous-intensity sports, sum of all METs-min/week from all vigorous-intensity physical activity, total METs-min/week from all physical activity compared with non-overweigh/non-obese. Categorized students to above or below 50% of WHtR also showed significant differences in METs-min/week from vigorous-intensity sports, sum of all METs-min/week from all vigorous-intensity and total METs-min/week from all physical activity. These findings indicate that students who are non-overweigh/non-obese and below 50% of WHtR expended more energy. On the other hand, there are no significant differences in other activity energy expenditure between all categorized groups. In all groups, the lowest energy expenditure was during martial art and cycling activities.

**Table 2:** Activity energy expenditure (METs-min/week) in different types of physical activity categorized by overweight/obese versus non-overweigh/non-obese and above or below 50% of waist/height ratio (W/HtR).

Variable	Non-overweigh/non-obese (N = 218)	Overweigh/obese (N = 253)	W/HtR < 0.50 (N = 243)	W/HtR ≥ 0.50 (N = 228)
METs-min/week from walking	347.8 (37.7)	327.8 (7.7)	348.6 (35.7)	324.800 (36.9)
METs-min/week from stair Stepping	119.1 (5.1)	106.2 (4.8)	117.9 (4.9)	106.0 (5.0)
METs-min/week from Jogging/running	491.2 (53.4)	398.1 (49.6)	495.2 (50.6)	383.6 (52.2)
METs-min/week from cycling	91.9 (21.7)	125.9 (20.1)	90.7 (20.5)	131.0 (21.2)
METs-min/week from swimming	165.3 (31.7)	178.2 (29.4)	157.6 (30.0)	187.7 (30.9)
METs-min/week from martial art	57.9 (26.4)	76.9(24.8)	90.0 (24.9)	44.8 (25.8)
METs-min/week from resistance training	223.0 (35.7)	182.3 (33.1)	212.6 (33.8)	188.9 (34.9)
METs-min/week from household activity	128.0 (21.2)	128.0 (19.7)	123.2 (20.1)	130.1 (20.7)
METs-min/week from moderate-intensity sports	211.9 (31.5)	214.1 (29.3)	212.4 (29.8)	213.9 (30.8)
METs-min/week from vigorous-intensity sports	871.1** (81.4)	519.1 (75.6)	832.2** (77.3)	521.9 (79.8)
Sum of METs-min/week from all moderate-intensity PA	641.1 (56.4)	631.2 (52.4)	629.4 (53.4)	642.6 (55.2)
Sum of all METs-min/week from all vigorous-intensity PA	2066.2* (139.5)	1622.7 (129.5)	2051.0* (132.1)	1590.3 (136.4)
Total METs-min/week from all PA	2707.3*(170.0)	2253.9 (157.8)	2680.4*(161.0)	2232.9 (166.2)

Significant values are in [bold].

Data are means and standard errors.

*Significantly different at < 0.05 level from the other groups using multivariate analyses test.

**Significantly different at < 0.01 level from the other groups using multivariate analyses test.

Tables 3 and 4 show screen time, sleep duration and eating habits of the students relative to overweight/obesity and WHtR categories; respectively. There were no significant differences between overweigh/obese and non-overweigh/non-obese students or between above or below 50% of WHtR neither in screen time nor sleep duration. The majority of the students (overweight/obese and non-overweigh/non-obese) watching screen above recommended cut-offs point. Only 18.3% of the non-overweigh/non-obese and 23.7% of overweight/obese met the recommended screen time guidelines of 3 h or less per day. Almost only one-third of non-overweigh/non-obese and overweight/obese sleep more than 8 h, whilst the majority of them sleep less than recommended hours (8 h). Similar findings were observed for above or below 50% of WHtR groups.

**Table 3:** Mean and proportion (%) of screen time, sleep duration and eating habits of the students relative to overweight/obesity levels.

Variable	Cut-offs	Non-overweigh/non-obese (N = 218)	Overweigh/obese (N = 253)	*p* values *
Screen time (hours/day)	Mean (SE)	6.3 (0.24)	6.1 (0.22)	0.501
	< 3 h	18.3	23.7	0.095
	> 3 h	81.7	76.3	
Sleep duration (hours/night)	Mean (SE)	6.9 (0.10)	6.8 (0.10)	0.218
	< 8 h	72.5	68.8	0.219
	8 + h	27.5	31.2	
Breakfast intake (day/week)	Mean (SE)	3.4 (0.16)	3.5 (0.18)	0.472
	1–2 days	49.2	49.3	0.426
	3–4 days	13.0	10.4	
	5 + days	37.8	40.3	
Fruit intake (day/week)	Mean (SE)	3.1 (0.16)	3.2 (0.15)	0.441
	1–2 days	43.6	46.2	0.083
	3–4 days	31.2	22.5	
	5 + days	25.2	31.2	
Vegetables intake (day/week)	Mean (SE)	3.7 (0.16)	3.6 (0.15)	0.703
	1–2 days	33.5	42.3	**0.025**
	3–4 days	28.0	18.2	
	5 + days	38.5	39.5	
Milk and dairy product intake (day/week)	Mean (SE)	4.4 (0.16)	4.3 (0.15)	0.436
	1–2 days	22.5	29.2	**0.043**
	3–4 days	28.0	19.0	
	5 + days	49.5	51.8	
Sugar sweetened drink intake(day/week)	Mean (SE)	3.5 (0.16)	3.8 (0.17)	0.095
	1–2 days	33.5	41.1	0.231
	3–4 days	26.1	22.5	
	5 + days	40.4	36.4	
Fast food intake (day/week)	Mean (SE)	3.1 (0.12)	2.9 (0.11)	0.193
	1–2 days	41.3	48.2	0.261
	3–4 days	39.0	32.4	
	5 + days	19.7	19.4	
Cake/donuts intake (day/week)	Mean (SE)	2.0 (0.12)	1.8 (0.12)	0.088
	1–2 days	65.1	73.5	0.125
	3–4 days	24.3	17.4	
	5 + days	10.6	9.1	
Chocolates/candy intake (day/week)	Mean (SE)	3.3 (0.14)	2.8 (0.13)	**0.006**
	1–2 days	38.1	49.0	**0.049**
	3–4 days	34.9	30.4	
	5 + days	27.1	20.6	
Energy drink intake (day/week)	Mean (SE)	0.78 (0.10)	0.79 (0.09)	0.987
	1–2 days	88.5	87.7	0.768
	3–4 days	8.7	8.3	
	5 + days	2.8	4.0	

Significant values are in [bold].

*Multivariate analyses test for the continuous variables and Chi Square test for testing the differences in the proportions between overweight/obese and non-overweight/non-obese adolescents.

**Table 4:** Means and proportion (%) of screen time, sleep duration and eating habits of the students relative to waist/height ratio category.

Variable	Cut-offs	W/HtR < 0.50 (N = 243)	W/HtR ≥ 0.50 (N = 228)	*p* values*
Screen time (hours/day)	Mean (SE)	6.3 (0.23)	6.0 (0.11)	0.333
	< 3 h	18.9	23.7	0.125
	> 3 h	81.1	76.3	
Sleep duration (hours/night)	Mean (SE)	6.9 (0.10)	6.8 (0.11)	0.466
	< 8 h	71.2	69.7	0.403
	8 + h	28.8	30.3	
Breakfast intake (day/week)	Mean (SE)	3.3 (0.18)	3.5 (0.18)	0.484
	1–2 days	49.4	49.1	0.623
	3–4 days	13.2	10.5	
	5 + days	37.4	40.4	
Fruit intake (day/week)	Mean (SE)	3.1 (0.14)	3.3 (0.15)	0.550
	1–2 days	43.6	46.5	0.120
	3–4 days	30.5	22.4	
	5 + days	25.9	31.1	
Vegetables intake (day/week)	Mean (SE)	3.7 (0.15)	3.6 (0.16)	0.755
	1–2 days	34.6	42.1	0.069
	3–4 days	26.7	18.4	
	5 + days	38.7	39.5	
Milk and dairy product intake (day/week)	Mean (SE)	4.5 (0.14)	4.2 (0.15)	0.268
	1–2 days	21.8	30.7	**0.013**
	3–4 days	28.0	18.0	
	5 + days	50.2	51.3	
Sugar sweetened drink intake(day/week)	Mean (SE)	3.6 (0.17)	3.8 (0.16)	0.347
	1–2 days	35.4	39.9	0.586
	3–4 days	24.7	23.7	
	5 + days	39.9	36.4	
Fast food intake (day/week)	Mean (SE)	3.1 (0.12)	2.9 (0.12)	0.181
	1–2 days	40.7	49.6	**0.044**
	3–4 days	40.7	29.8	
	5 + days	18.5	20.6	
Cake/donuts intake (day/week)	Mean (SE)	2.1 (0.12)	1.7 (0.12)	**0.013**
	1–2 days	64.6	75.0	**0.042**
	3–4 days	24.7	16.2	
	5 + days	10.7	8.8	
Chocolates/candy intake (day/week)	Mean (SE)	3.4 (0.13)	2.7 (0.13)	**< 0.001**
	1–2 days	36.2	52.2	**0.002**
	3–4 days	36.6	28.1	
	5 + days	27.2	19.7	
Energy drink intake (day/week)	Mean (SE)	0.83 (0.09)	0.74 (0.10)	0.477
	1–2 days	87.7	88.6	0.921
	3–4 days	8.6	8.3	
	5 + days	3.7	3.1	

Significant values are in [bold].

*Multivariate analyses test for the continuous variables and Chi Square test for testing the differences in the proportions between overweight/obese and non-overweight/non-obese adolescents.

In terms of eating habits, the mean of breakfast intake for non-overweigh/non-obese and overweight/obese were 3.4 (0.16) and 3.5 (0.18) day/week, respectively. No significant difference in the mean of breakfast intake was observed between the two groups. There was also no significant difference in the mean of fruit intake between non-overweigh/non-obese and overweight/obese groups. In addition, no significant difference for the mean of breakfast intake or fruit intake between above and below 50% of WHtR groups. Nearly half of overweight/obese and non-overweigh/non-obese as well as above and below 50% of WHtR groups ate breakfast and fruits only 1–2 days per week.

There was no significant difference in the mean of vegetable intake between non-overweigh/non-obese and overweight/obese groups. However, there was a significant association between vegetable intake and obesity based on BMI cut-offs. The overweight/obese group were less likely to consume vegetables than were non-overweigh/non-obese group (33.5% of the non-overweigh/non-obese group consume vegetable 1–2 days/week, whilst 42.3 of the overweight/obese group consume vegetable 1–2 days/week).

There were significant associations between the intake of milk and dairy product and obesity. The percentage of the non-overweigh/non-obese group who consumed milk and dairy products 1–2 days/week was 22.5% versus 29.2 of the overweight/obese group. Based on waist/height ratio category, the consumption of milk and dairy products were 21.8% and 30.7% in below and above 50% of WHtR groups; respectively.

There were significant associations between fast food intake and WHtR. The above 50% of WHtR groups reported more frequent intake (+ 5 days/week) of fast food (20.6%) than the below 50% of WHtR groups (18.5%).

There were significant differences in the mean of chocolates/candy intake between non-overweigh/non-obese and overweight/obese groups as well as between above and below 50% of WHtR groups. There were also significant associations between intake of chocolates/candy and obesity. Furthermore, the results show significant associations between cake/donuts intake and obesity based on WHtR category.

The majority of students in all groups consume energy drinks only 1–2 days per week, and no significant association between energy drinks and obesity.

Table 5 shows the results of multiple regression analyses for the prediction of total activity energy expenditure, sum of moderate or vigorous physical activities. The entered variables explained 8.1%, 6.8% and 6.4% (R^2^) of the variance in total activity energy expenditure, sum of vigorous intensity physical activity and sum of moderate intensity physical activity; respectively. As determined by a stepwise procedure, fruit intake is a susceptible predictor of total activity energy expenditure, sum of moderate and vigorous physical activities while waist circumference is a good predictor variable only for total activity energy expenditure and vigorous physical activities. Interestingly, sugar-sweetened drinks intake was predictor factor of sum of moderate intensity physical activity.

**Table 5:** Results of multiple regression analyses for the prediction of total activity energy expenditure, sum of moderate or vigorous physical activities.

Dependent variable	Predictor variables*	Standardized coefficient (Beta)	*p* value	R^2^
Total activity energy expenditure (METs-min/week)	Fruit intake	0.243	< 0.001	0.081
	Waist circumference	− 0.153	0.001	
Sum of vigorous intensity physical activity (METs-min/week)	Fruit intake	0.202	< 0.001	0.068
	Waist circumference	− 0.167	< 0.001	
Sum of moderate intensity physical activity (METs-min/week)	Fruit intake	0.222	< 0.001	0.064
	Sugar-sweetened drinks intake	− 0.101	0.025	

*Entered variables included age, average screen time, average sleep duration, breakfast intake, Fruit intake, vegetables intake, milk/dairy products intake, sugar-sweetened drinks intake, fast food intake, cake/donuts intake, chocolate/candy intake, body mass index, Waist circumference and waist/height ratio.

## Discussion

Over the last few decades, Saudi Arabia has experienced rapid industrial and economic growth and technological transformation along with huge lifestyle changes. These changes in lifestyle habits including physical activity, eating and sleep habits accompanied by rapid increase in obesity prevalence. A better understanding of the associations between obesity and lifestyle habits is essential to manage obesity prevalence. The present study aimed to provide more information regarding physical activity, sedentary behavior and eating habits and apparent obstacles to a healthy lifestyle in adolescents students in Saudi Arabia.

It has been documented that adolescent obesity is associated with suboptimal physical activity levels, poor fitness and excessive time in sedentary behaviors^22^. Our findings provide evidence that high prevalence of sedentary behaviors and physical inactivity among adolescent students is associated with obesity. An earlier study in Saudi Arabia showed that inadequate physical activity was associated with obesity and the lack of exercise was a major risk factor for obesity among adolescents^23^. The high prevalence of physical inactivity and sedentary behaviors was cosidered as a major public health concern among Saudi adolescents^19^.

Al-Hazzaa et al.^19^ reported that the total METS-min/Week for male adolescents to be 2613.2, however, in our study it was 2480.6. Both moderate -intensity and vigorous -intensity activities were lower in our study compared with Al-Hazzaa et al.^19^ especially the moderate -intensity activity. The Saudi Ministry of Health is planning to reduce the rate of overweight and obesity among school children through the implementation of various measures such as promoting physical activity as well as healthy eating habits. However, these measures will not be effective without understanding the actual factors associated with obesity among school children. Studies on obesity among adolescents in Saudi Arabia are well documented but the habits associated with a high prevalence of obesity among school age group were not established. Understanding the influence of lifestyle-related variables on overweight and obesity among Saudi adolescents are important for developing public policies to decrease the obesity. Our findings suggest a need for further improvement in strategies promoting healthier eating habits. Schools provide many opportunities to positively influence physical activity, diet, and weight management behaviors in the educational setting. Ideally, if school students make positive changes in physical activity and dietary practices, these changes could persist into adult and create a healthy future generation. It is encouraged to create a culture of awareness of the problems allied with obesity in Saudi youth to motivate them to lead a healthy lifestyle.

The present study reveals that physical inactivity was associated with obesity. These findings are in a line with findings of Patrick et al.^24^, Janssen et al.^25^, Moliner-Urdiales et al.^26^ and Boutelle et al.^27^. Moliner-Urdiales et al.^26^ examined the association of physical activity with central body fat and found that adolescents who engaged in moderate level of physical activity for 60 min a day had lower levels of total body fat and central body fat.

The high prevalence of unhealthy eating habits is one of the major health concerns among Saudi adolescents^19^. Our findings provide more evidence on the the prevalence of unhealthy eating habits among the students. Some eating habits were found in the current study to be associated with obesity among the adolescents. These finding are in agrrement with several studies which clearly indicating that skipping breakfast is the most important risk factor for overweight and obesity^28,29^. The risk of obesity was associated with increased frequency of eating potatoes or sweets among adolescents. These findings are consisted with Chacar and Salameh^30^. In contrast, eating vegetables was associated with a lower risk of overweight and obese^31^.

Fast food consumption was significantly associated with overweight and obesity in a children and adolescents^31,32^. In the present study, we found that high consumption of fast foods was allied with increased risks of obesity. Sugar sweetened beverages were widely contributing to the overweight and obesity^33^. Daily consumption of fruit and vegetables are an important indicator of a healthy diet and the beneficial effect of lowered BMI has been well documented^34^. The present study findings suggest that there are multiple factors can cause obesity. Our findings show that fruit intake and sugar-sweetened drinks were the closest estimates of moderate-intensity physical activity; with the lowest standard error of estimation which means fruit intake and sugar-sweetened drinks and are more sensitive to predicting moderate-intensity physical activity. In addition, fruit intake and waist circumference were the closest estimates of total activity energy expenditure as well as vigorous -intensity physical activity.

Lack of physical activity is a risk factor for high BMI in adolescent. Findings from a cross-sectional survey of adolescents from many countries demonstrated that physical inactivity and high television viewing times were allied with overweight^30^. The present study findings showed that that vigorous-intensity physical activity was inversely associated with adolescent obesity is also widely supported in the literature^26^. Our findings suggest that public health education, physical activity and healthy eating habits are essential to minimize the burden of overweight and obesity. In this regard, a role for school and family is vital with the aim of placing young people towards better eating habits, exercise behavior and quality of life.

There are few limitations of this study. First, the sample was representative of one city of Saudi Arabia and sampling methodology was voluntary self-selection. This is a cross-sectional design provide a snapshot of current status. The data is based on self-reported students’ recall by a questionnaire, and energy intakes was not measured. However, this study described the lifestyle behaviors and provides valuable and significant information for the adolescent health. The results of this study are strengthened by a reasonable sample size, collectively examined several lifestyle factors in a Saudi adolescents, using a validated and comprehensive physical-activity questionnaire, employing metabolic equivalents for calculating energy expenditure from physical activity.

## Conclusion

This study demonstrated that the adolescents show lower levels of physical activity and spend more time in front of the television and computers. They also had higher prevalence of inadequate consumption of healthy foods, fruit and vegetable. These results highlight the importance of establishing education policy about the importance of adopting healthy behaviors in adolescence. Emphasis should be given to implementing policies to education the people for physical activity, healthy diet, thereby reducing the future risk of chronic diseases.

## Data Availability

All data generated or analyzed during current study are included in this published article. Additional data are available from the corresponding author on reasonable request.
